# Supplementary material for: Clinical features of chronic cluster headache based on the third edition of the International Classification of Headache Disorders: A prospective multicentre study
Source: PLoS One. 2019 Aug 26;14(8):e0221155. doi: 10.1371/journal.pone.0221155 (PMC6709915; doi:10.1371/journal.pone.0221155)
Supplement: S1 Table — (DOCX) [file pone.0221155.s001.docx]

**S1 Table. Clinical features of participants with secondary chronic cluster headache and episodic chronic cluster headache.**

|  | | Secondary CCH,  n=6 | ECH,  n=176 | *p*-value |
| --- | --- | --- | --- | --- |
| Age (years) | | 44.5 (30.0–59.8)^*^ | 39.0 (32.0–45.0)^*^ | 0.352 |
| Women, n (%) | | 0 (0.0) | 24 (14.0) | 0.845 |
| Body Mass Index | | 24.3 (22.0–28.2)^*^ | 23.8 (22.0–25.7)^*^ | 0.524 |
| Attack frequency per day | | 2.8 (0.5–7.0)^*^ | 1.1 (1.0–3.0)^*^ | 0.406 |
| VAS, median | | 9.0 (8.6–10.0)^*^ | 9.0 (8.0–10.0)^*^ | 0.779 |
| Attack duration (minutes) | | 75.0 (52.5–180.0)^*^ | 60.0 (60.0–120.0)^*^ | 0.882 |
| Cluster period (weeks) | | 8.0 (1.8–47.3)^*^ | 4.00 (3.0–7.50)^*^ | 0.882 |
| Total number of bouts | | 16.5 (5.0–57.0)^*^ | 7.00 (3.0–12.0)^*^ | 0.119 |
| Years after CH onset | | 5.5 (2.5–7.3)^*^ | 10.0 (5.0–16.0)^*^ | 0.041 |
| Age of onset of CH | | 40.5 (24.0–50.0)^*^ | 24.0 (18.0–32.8)^*^ | 0.060 |
| Change of pain side during a single bout period, n (%) | | 1 (16.7) | 9 (5.2) | 1.000 |
| Currently smoking, n (%) | | 4 (66.7) | 79 (45.9) | 0.025 |
| Diurnal rhythmicity, n (%) | | 0 (0.0) | 90 (51.1) | 0.011 |
| Migraine, n (%) | | 1 (16.7) | 19 (10.8) | 1.00 |
| Location, n (%) | |  |  |  |
|  | Orbital | 6 (100.0) | 79 (45.9) | 0.072 |
|  | Temporal | 5 (83.3) | 90 (53.3) | 0.241 |
|  | Supraorbital | 5 (83.3) | 19 (10.8) | 1.000 |
| Accompanying symptoms, n (%) | |  |  |  |
|  | Conjunctival injection and/or lacrimation | 6 (100.0) | 152 (88.4) | 1.000 |
|  | Nasal congestion and/or rhinorrhoea | 1 (16.7) | 110 (64.0) | 0.068 |
|  | Eyelid oedema | 3 (50.0) | 50 (29.1) | 0.182 |
|  | Forehead and facial sweating | 2 (33.3) | 54 (31.4) | 1.000 |
|  | Miosis and ptosis | 0 (0.0) | 6 (3.5) | 1.000 |
|  | Restlessness/ agitation | 1 (16.7) | 40 (23.3) | 1.000 |
| Headache Impact Test-6 | | 72.5 (65.8–76.5)^*^ | 69.0 (64.0–76.0)^*^ | 0.491 |
| Severe impact of headache (Headache Impact Test-6 score ≥ 60) | | 6 (100.0) | 143 (81.3) | 0.591 |
| Perceived Stress Scale | | 7.5 (0.8–9.0)^*^ | 7.0 (4.0–8.0)^*^ | 0.220 |
| EQ-5D-3L | | 0.91 (0.68–1.00)^*^ | 0.91 (0.82–1.00)^*^ | 0.966 |
| Patient Health Questionnaire-9 | | 4.50 (0.0–8.8)^*^ | 5.0 (3.0–11.0)^*^ | 0.326 |
| Generalized Anxiety Disease-7 | | 5.0 (0.8–9.0)^*^ | 7.0 (3.0–12.0)^*^ | 0.220 |

CCH, chronic cluster headache; CH: cluster headache; ECH, episodic cluster headache; VAS, visual analogue scale; EQ-5D-3L, The 3-level version of European quality of life-5 dimensions; *: median (25%–75% interquartile range)
