# Supplementary material for: Clinical features of chronic cluster headache based on the third edition of the International Classification of Headache Disorders: A prospective multicentre study
Source: PLoS One. 2019 Aug 26;14(8):e0221155. doi: 10.1371/journal.pone.0221155 (PMC6709915; doi:10.1371/journal.pone.0221155)
Supplement: Attachment — Submitted filename: author response letter_R1.docx [file pone.0221155.s002.docx]

Author Response Letter

We thank the reviewers for their constructive comments. We believe our paper has been improved based on the Reviewers’ comments. In this letter, we have provided a point-by-point response to each comment below. Textual changes based on the Reviewers’ comments are tracked in the revised manuscript. We hope our paper can meet the journal’s requirements.

Reviewer #1

1. The definition of chronic cluster headache has been changed in the last ICHD3 classification, increasing the time for relapse period from 1 to 3 months. The authors tested of the Korean cluster headache registry the effect of this modification in order to test the new definition and the clinical features of the CCH.

**Response**: We thank the reviewer for his/her time and efforts in reviewing our paper.

2. The most relevant observation is that the number of patients with CCH doubles with the new criteria.

**Response**: We agree with the reviewer that this is the major finding of our study. The latest ICHD-3 criteria broadened the spectrum of CCH without compromising its functional impact.

3. The number of patients with CCH vs ECH are much less (176 vs 12) but the authors were able, with the statistical correction to still be able to find some significant diferences between the two populations. In particular they found out that the patients with CCH has higher age and lower CH history vs patients with ECH, they have less nasal congestion and rhinorrhea, and have more comorbidity with migraine. Interestingly the authors did not find any difference in the population reclassified as CCH with the new criteria, meaning that the patients are probably right classified with the new criteria. They observed how smoke habit may lead CH to become chronic lathough to clearly significant (probably due to the small numeber of pts). The limits of the study where well stated.

**Response**: We appreciate the reviewer for properly summarizing our findings and giving us constructive comments.

4. It would be interesting in the discussion to speculate if there might be a possible role of chronic pain networks dysfunction in both conditions (Colombo B et al Neurol SCI 2015).

**Response**: Thank you for the insightful suggestion. We have added discussions regarding the role of pain networks in CH chronification. **(Line 307 – 314 in the track-changes version)**

Reviewer #2: This is the multi-center, prospective study to investigate the clinical feature of chronic cluster headache using the new definition in hospitala in south korea. Overall, manuscript is well written. I cannot identify the major of this study.

**Response**: We thank the reviewer for his/her time and efforts in reviewing our paper and constructive comments. We are pleased that the reviewer was satisfied with our paper.

Reviewer #3:

1. Please discuss in the limitations the types of biases the findings of this study may be influenced by

**Response**: We thank the reviewer for his/her time and efforts in reviewing our paper. The classification of ECH and CCH was based on patients’ description of their clinical courses, so recall bias may be present. Our study may also have type II error because the number of patients with CCH was small. We have added these information in the Limitations section. **(Line 316 – 318, 328 – 329 in the track-changes version)**

2. The limitations of the study particularly in regards to the validation of diagnosis needs to be more robust.

**Response**: Thank you for this comment. In our study, the validation of the ICHD-3 criteria was based on the comparison between 6 and 12 patients. This may be affected by biases because the number of patients was small, and the two groups were not completely independent to each other. We have added this in the Limitation section. **(Line 332 – 334 in the track-changes version)**

3. Some revision of the English language is needed.

3-1. “Diagnosis of ECH (code 3.1.1) and CCH (code 3.1.2) were based on criteria” is not correct in grammar.

**Response**: Thank you for pointing this out. The expressions have been revised. **(Line 96 – 97, 108 – 112 in the track-changes version)**

3-1. “huge” should be avoided in academic writing.

**Response**: Thank you for the suggestion. This expression has been also revised. **(Line 278 in the track-changes version)**
